# Examining the Representation of South Asian Populations in Substance Addiction Research: A Bibliometric Analysis From 2014 to 2024

**DOI:** 10.7759/cureus.93126

**Published:** 2025-09-24

**Authors:** Saswat Sahoo, Khushi Singh, Teresa Fong, Asmaa Basonbul

**Affiliations:** 1 Department of Medicine, University of Alberta, Edmonton, CAN; 2 Department of Psychology, University of Toronto, Toronto, CAN; 3 Department of Hematology, Faculty of Medicine, Umm Al Qura University, Makkah, SAU

**Keywords:** bibliometric analyis, publication trends, south asian, substance addiction, substance use disorder

## Abstract

Substance addiction is a major global health concern, yet South Asian populations remain underrepresented in research, limiting understanding of how addiction affects these communities. Therefore, this study aimed to evaluate substance addiction research productivity in South Asian populations through a 10-year bibliometric analysis (2014-2024).

A systematic PubMed search identified 1,320 research publications that met the inclusion criteria. Extracted data included annual publication counts, article type, five-year journal impact factor (JIF), citation counts, and country of publication. South Asian populations were examined both as a whole and as specific subgroups from Afghanistan, Bangladesh, Bhutan, India, the Maldives, Nepal, Pakistan, and Sri Lanka.

Annual publications showed a significant upward trend (slope=3.73 studies/year, p=0.016), increasing from 124 in 2014 to 164 in 2024. The highest number of studies was conducted on the Indian population (n=873; 66.1%), which was also the only group to show a significant growth trend (slope=3.13 studies/year, p=0.03). Additionally, most publications were original research articles (58.9%), with a mean five-year JIF of 4.87 and an average of 30.5 citations per article. Populations from Afghanistan and India had the highest values for these metrics, while the rest remained underrepresented. Furthermore, India (n=494) and the United States (n=332) were the top countries of publication, together producing 63% of all research. Notably, six of the top 10 publishing countries were outside South Asia.

In conclusion, research on substance addiction in South Asian populations has grown significantly over the past decade but remains heavily skewed toward India, with other groups underrepresented in both research quantity and quality. Through identifying these trends, this study highlights critical gaps and priorities for more equitable research.

## Introduction and background

Substance addiction, otherwise known as substance use disorder (SUD), is a medical condition defined by the persistent and maladaptive consumption of substances, such as alcohol or drugs [1]. Over the last few decades, substance addiction has emerged as a significant public health concern, with drug-related mortality rates increasing by over 600% worldwide [2]. In South Asia specifically, more than 66 million people are estimated to use psychoactive substances, with high prevalence of alcohol and opioid use reported in several countries [3]. Despite this considerable burden, the majority of the work has focused on Western populations [4,5]. In contrast, South Asian populations, both within South Asia and in diaspora communities across North America, Europe, and elsewhere, remain underrepresented in addiction research. This underrepresentation limits our understanding of how substance addiction affects South Asian groups, whose cultural norms and socioeconomic conditions differ significantly from those of Western groups. Factors such as stigma surrounding mental health [6], limited access to addiction treatment services [7], and reliance on traditional healing practices [6] can influence addiction trajectories in these populations. For example, in many South Asian communities, substance use is often seen as a reflection of weak character, a source of dishonor for the family, or a ‘sin’ that violates religious codes [8]. Such cultural stigma may prevent individuals from seeking treatment and contribute to the underreporting of substance use problems in the region.

While substance addiction studies have been conducted among South Asians, very few have analyzed the overall research activity in this field. One such study [5] examined publication trends, though it was limited to the years 1995-2014 and did not disaggregate data by specific South Asian groups. Another study [1] examined this topic more recently, from 1995 to 2023, though it focused on Asian Americans rather than specifically South Asians. As a result, the current state and evolution of research productivity in South Asian populations remain poorly characterized. Understanding these dynamics is essential for assessing scientific progress, identifying research gaps, and guiding future priorities to ensure equitable representation in global research.

Therefore, our study aimed to build on prior bibliometric analyses by providing the first up-to-date, comprehensive evaluation of research productivity on substance addiction in South Asian populations over the past decade (2014-2024). This timeframe was selected to capture the most recent trends following earlier analyses that ended in 2014. Research productivity was evaluated through annual publication output, article types, journal impact factors, citation counts, and countries of publication. We also conducted sub-analyses to examine trends within specific South Asian populations, defined by the United Nations as including Afghanistan, Nepal, Pakistan, India, Sri Lanka, Bhutan, Bangladesh, and the Maldives [9].

## Review

Methods

Search Strategy

We conducted this bibliometric study using the PubMed database to retrieve all relevant research publications on substance addiction in South Asian populations in the past 10 years (2014-2024). As we used publicly available data, informed consent and ethics approval were not required. We limited our search to one database, as the aim of our study was to capture bibliometric trends rather than exhaustively retrieve every article. PubMed was chosen for its comprehensive, freely accessible coverage and rigorous indexing standards, including controlled vocabulary terms (MeSH) and detailed publication type classifications. A systematic search strategy (Table 1) was employed on July 29th, 2025, using keywords for substance addiction [10] and South Asia [11] that were informed by prior research. This search strategy yielded a total of 6,639 publications.

**Table 1:** Details of the PubMed search strategy for article selection

Concept	Search Terms
Substance Addiction	(substance related disorders[MeSH Terms] OR alcohol*[Title/Abstract] OR "substance use*"[Title/Abstract] OR "substance abuse*"[Title/Abstract] OR "substance dependenc*"[Title/Abstract] OR "substance misus*"[Title/Abstract] OR "substance addiction*"[Title/Abstract] OR "substance disorder*"[Title/Abstract] OR "drug use*"[Title/Abstract] OR "drug abuse*"[Title/Abstract] OR "drug dependenc*"[Title/Abstract] OR "drug misus*"[Title/Abstract] OR "drug addiction*"[Title/Abstract] OR "drug disorder*"[Title/Abstract] OR "illicit drug*"[Title/Abstract] OR "illicit substance*"[Title/Abstract] OR "recreational drug*"[Title/Abstract] OR "illegal substance*"[Title/Abstract] OR "illegal drug*"[Title/Abstract])
South Asia	("South* Asia*"[Title/Abstract] OR Afghan*[Title/Abstract] OR Nepal*[Title/Abstract] OR Pakistan*[Title/Abstract] OR India*[Title/Abstract] OR "Sri Lanka*"[Title/Abstract] OR Bhutan*[Title/Abstract] OR Bangladesh*[Title/Abstract] OR Maldiv*[Title/Abstract])
Final Search	#1 AND #2, from 2014-2024

Eligibility Criteria

We then applied PubMed’s search filters for article type to limit results to peer-reviewed research publications to evaluate true research productivity, which included either original research or review articles. Filters for original research included Adaptive Clinical Trial, Case Reports, Clinical Study, Clinical Trial, Clinical Trial Phase I, Clinical Trial Phase II, Clinical Trial Phase III, Clinical Trial Phase IV, Comparative Study, Controlled Clinical Trial, Equivalence Trial, Evaluation Study, Multicenter Study, Observational Study, Pragmatic Clinical Trial, Randomized Controlled Trial, Twin Study, and Validation Study. Filters for review articles included meta-analysis, network meta-analysis, review, scoping review, and systematic review. We did not exclude any articles based on the language of publication.

Study Selection

**Figure 1:**
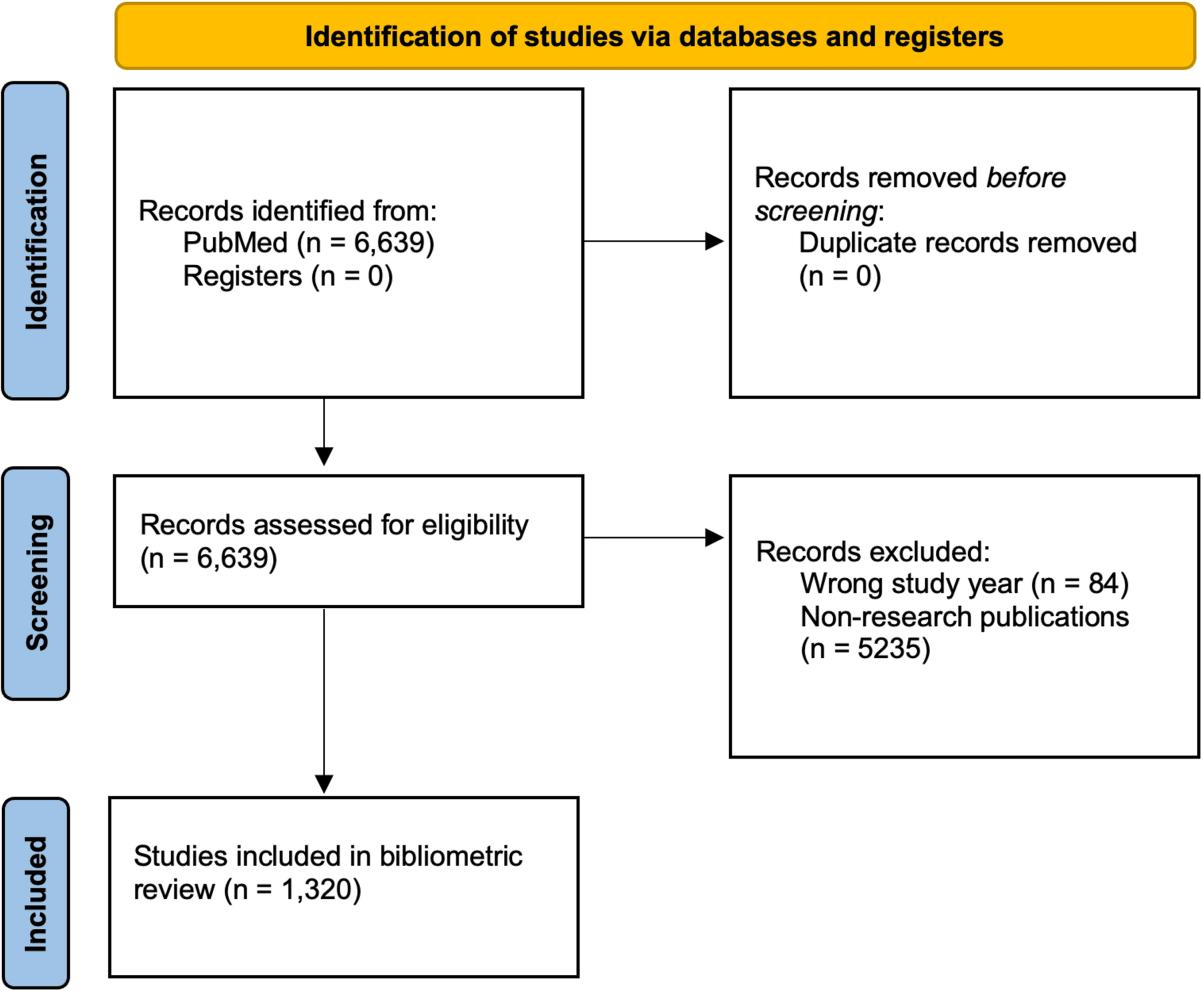
PRISMA flow diagram of the study selection process

Ultimately, this process led to the selection of 1,320 research publications, as illustrated using the Preferred Reporting Items for Systematic Reviews and Meta-Analyses (PRISMA) flowchart (Figure 1). These papers were further reviewed and analyzed to produce the findings reported in this study.

Data Extraction

We assessed the research productivity of this field by determining various metrics for each research article: annual research output (number of publications per year), types of research publications (number of original research vs. review articles), five-year journal impact factor (JIF), citation counts (number of times the work was cited), and country of publication. The five-year JIF was determined through the official Clarivate Journal Citation Reports, while the number of citations was determined using the Web of Science Core Collection. The country of publication was determined based on the first author’s affiliation, indicating where the majority of the work was conducted. For many of the analyses, we also stratified by “Population of Interest,” which was determined by identifying the research population studied in each through reviewing titles and abstracts. Populations of interest included those from Afghanistan, Nepal, Pakistan, India, Sri Lanka, Bhutan, Bangladesh, and/or the Maldives, whether residing within South Asia or as part of diaspora communities abroad. This allowed us to more closely examine the research trends for specific South Asian populations, beyond simply considering them as a single group. To ensure accuracy, three independent reviewers collected the data and cross-verified the results.

Risk of Bias and Quality Assessment

As this was a bibliometric analysis aimed at evaluating research output rather than the quality of individual articles, we did not conduct a formal risk of bias assessment. Similarly, no meta-analysis or meta-regression was performed, given the focus on research productivity rather than clinical outcomes.

Statistical Analysis

A linear regression model was used to estimate the slope and assess statistical significance. Linear regression was selected, as it is widely used in bibliometric analyses to assess temporal publication trends, and more complex methods were not warranted given the limited dataset (10 annual time points). All statistical analyses and visualizations were conducted using R version 4.4.0. A p-value less than 0.05 was considered statistically significant. No adjustments for multiple comparisons were made, as this was an exploratory study.

Results

Annual Research Output

**Figure 2:**
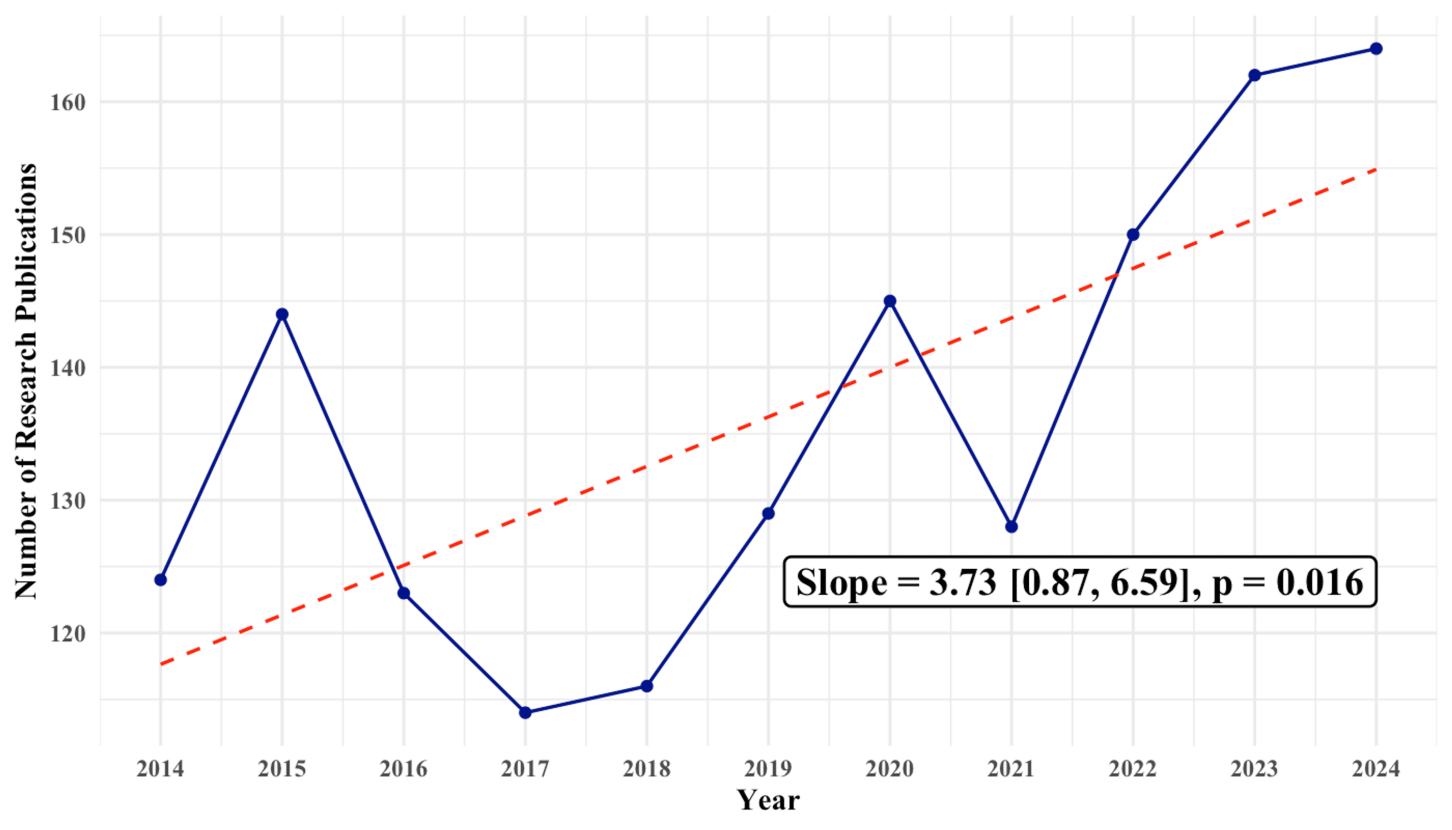
Annual trend in research publications Each dot represents the number of research publications for a given year. A linear regression model was used to generate a trendline (dashed) to estimate the slope and assess statistical significance. 95% confidence intervals are reported in square brackets. The image is created by the author.

The annual number of research publications showed a statistically significant upward trend (slope=3.73 studies/year, p=0.016), increasing from 124 in 2014 to 164 in 2024 (Figure 2). A temporary low was observed in 2017 (n=114), with a brief dip again in 2021.

**Figure 3:**
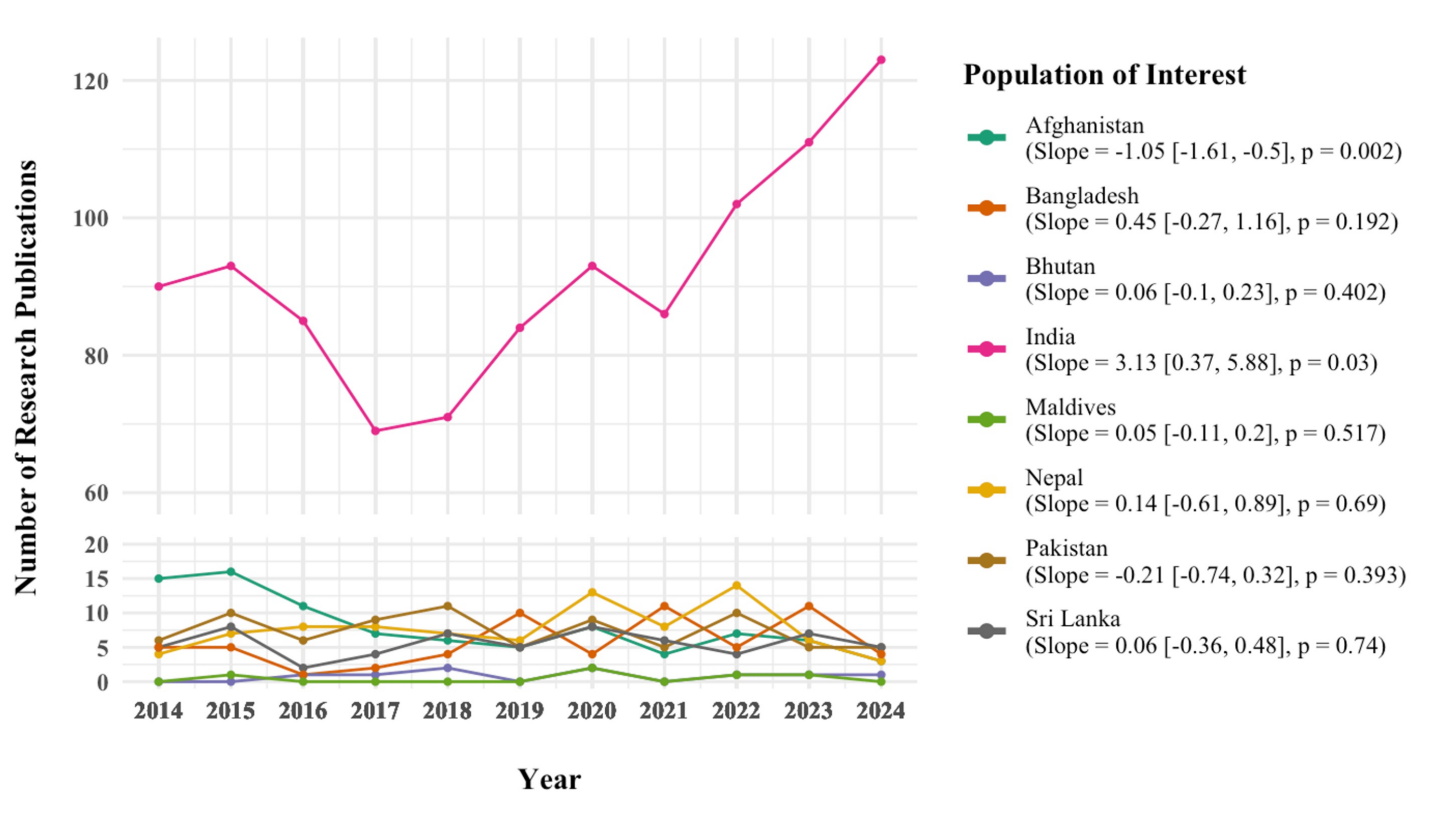
Annual trend in research publications within specific South Asian populations of interest Each dot represents the number of research publications for a given year. For each population of interest, a linear regression model was used to estimate the slope and assess statistical significance. A total of 95% confidence intervals are reported in square brackets. The image is created by the author.

When examining the number of studies conducted on specific South Asian populations (Figure 3), India accounted for the largest volume of publications and showed a significant increase (slope=3.13 studies/year, p=0.03). Research on Afghanistan significantly declined (slope=−1.05 studies/year, p=0.002), while other populations showed no significant changes.

Types of Research Publications

There were a total of 1,320 research publications on the South Asian population as a whole (Table 2). Within the specific populations of interest, the largest number of studies focused on the Indian population (n=873, 66.1%), and the fewest examined the Maldivian population (n=5, 0.4%). Overall, most publications were original research articles (58.9%) compared to review articles (41.1%); a trend was also observed for studies involving populations from India, Nepal, Sri Lanka, and Bangladesh. Other populations showed more balanced proportions.

**Table 2:** Types of research publications across South Asian populations Each value represents the total number of research publications across the study period from 2014 to 2024.

Population of Interest	Original Research Articles	Review Articles	Total research publications
South Asia	778	542	1,320
Afghanistan	40	39	79
Nepal	52	26	78
Pakistan	36	40	76
India	536	337	873
Sri Lanka	34	20	54
Bhutan	3	5	8
Bangladesh	34	23	57
Maldives	2	3	5

Journal Impact Factor and Citation Counts

The mean five-year JIF was 4.87, with an average of 30.5 citations per publication (Table 3). Afghanistan-focused studies had the highest averages for both JIF and citations, followed by India. Bhutan had the lowest averages. The highest JIF (105) was observed for studies on Afghanistan and India, and the greatest number of citations (3,799) was recorded for a publication on India.

**Table 3:** Journal impact factor and citation metrics across South Asian populations JIF: Journal Impact Factor

Population of Interest	5 Year JIF		Times Cited	
	Mean	Range	Mean	Range
South Asia	4.87	0 – 105	30.5	0 – 3799
Afghanistan	6.29	1 – 105	28.4	0 – 165
Nepal	3.54	1 – 12	17.7	0 – 114
Pakistan	3.59	1 – 24	27.2	0 – 608
India	4.67	0 – 105	28.3	0 – 3799
Sri Lanka	4.07	1 – 28	24.3	0 – 165
Bhutan	2.66	1 – 5	13.0	0 – 48
Bangladesh	4.61	1 – 24	18.8	0 – 150
Maldives	3.38	3 – 5	19.5	1 – 48

Top Countries of Publication

Our analysis of the top 10 countries of publication (Table 4) showed that India produced the highest number of research publications (n=494), followed by the United States (n=332). Together, these two countries accounted for a large share (approximately 63%) of the total research output. While other South Asian countries contributed, their output remained relatively modest. Notably, six of the top 10 contributing countries were outside South Asia, which included the United States, the United Kingdom, Australia, Malaysia, China, and Canada. This distribution suggests that much of the research on South Asian populations was conducted by institutions outside the region.

**Table 4:** Top 10 countries by number of research publications Country of publication refers to the first author’s country of affiliation.

Country of publication	Number of research publications
India	494
United States	332
United Kingdom	71
Australia	41
Pakistan	41
Nepal	37
Bangladesh	28
Sri Lanka	26
Malaysia	21
China	20
Canada	19

Discussion

This bibliometric analysis provides the first comprehensive overview of research productivity on substance addiction in South Asian populations over the past decade. We examined various South Asian communities, which included Afghanistan, Nepal, Pakistan, India, Sri Lanka, Bhutan, Bangladesh, and the Maldives. Our findings indicated an overall upward trend in the output of research publications, suggesting a growing academic interest. However, the distribution of this growth and the nature of publications across specific South Asian groups revealed notable disparities and underrepresentation within the field.

Trends in the Number of Research Publications

One of the key findings of our analysis was the statistically significant upward trend in the annual number of publications from 2014 to 2024. With a slope of 3.73 studies per year, this growth suggests increasing scholarly attention to substance addiction among South Asian populations. This trend mirrors global patterns of increasing addiction-related research, likely driven by growing public health concerns and increasing rates of substance abuse. For instance, a recent bibliometric study on global drug addiction research from 2015 to 2024 also reported a consistent increase in publication numbers [12]. However, our results showed a dip in publication output in 2017 and a brief decline again in 2021. The 2017 drop may reflect a temporary shift in research funding priorities or political instability in parts of South Asia, while the 2021 decrease likely corresponds to broader disruptions from the COVID-19 pandemic that affected scientific output across many fields globally [13]. These short-term dips did not disrupt the long-term upward trend.

The increasing publication trend is promising, but a closer look at the specific populations of interest reveals striking disparities. The majority of the studies (66.1%) centered on the Indian population, which was the only group to show a statistically significant increase in publication numbers over the last decade. Conversely, research focused on Afghanistan showed a statistically significant decline over the decade, which is particularly concerning given its historically high rates of opiate production and consumption [14]. For the other South Asian populations, publication trends showed no statistically significant change and remained low in number. The Maldives, in particular, accounted for only 0.4% of studies, a strikingly low number. Together, these findings suggest that the overall growth in substance addiction research activity on South Asian populations is largely driven by studies on India, leading to the underrepresentation of other communities in this field. These results align with earlier reviews that also identified India as the most frequently studied South Asian country in mental health research [11,15]. This may be attributable to India’s large demographic and relatively robust academic infrastructure in South Asia, as well as the country’s expanding public health efforts on substance use. For example, India's Ministry of Social Justice and Empowerment and the National Drug Dependence Treatment Centre have increased funding and surveys in recent years [16], which may correlate with the rise in scholarly output.

Patterns in Quality of Research Publications

Our analysis revealed that original research articles made up the majority (58.9%) of publications compared to review articles (41.1%), indicating that empirical investigation rather than synthesis of existing knowledge forms the backbone of this research area. This is encouraging, as primary data collection within South Asian populations is essential to developing evidence-based policies and interventions. This aligns with trends observed in other areas of health science, where original research studies typically outnumber review articles [17], reflecting a healthy engagement with empirical investigation in substance addiction research. In addition, a greater proportion of original studies were conducted in countries such as India, Sri Lanka, and Bangladesh, likely due to more advanced research infrastructure that supports primary data collection.

The mean five-year Journal Impact Factor (JIF) of 4.87 and average citation count of 30.5 suggest a moderate impact for most publications. Interestingly, studies focusing on Afghanistan, despite its declining frequency, had the highest mean five-year JIF and citation counts. This paradoxical finding may be explained by the global relevance of opioid production and trafficking in Afghanistan [14], which often draws the attention of high-impact journals and international researchers. It is also possible that the limited studies done on Afghanistan were produced through collaborations with researchers from high-resource institutions abroad, thereby enhancing the visibility and impact of the work. Additionally, India-focused publications had the highest citation counts (3,799 citations) and five-year JIF (105), which further underscores India’s prominent research activity in this field. In contrast, the other South Asian populations had lower JIF and citation counts, reflecting limited scholarly attention. These disparities highlight the persistent inequities in global health research, where smaller or less-resourced countries struggle to gain recognition despite potentially urgent local health issues [18]. Addressing these disparities will require greater investment in regional research consortia, targeted funding schemes to support locally led projects, and training initiatives that build sustainable research capacity within South Asia.

Geographic Sources of Research Production

A notable and somewhat concerning finding from our analysis of the top 10 countries of publication was that the majority of the research on South Asian populations was produced by institutions outside the region, such as the United States, the United Kingdom, Australia, Canada, Malaysia, and China. Aside from India, research output from all other South Asian countries was relatively low, despite their significant public health burdens related to substance use. A previous bibliometric review in global health has similarly noted the disproportionate role of foreign institutions in conducting research on countries other than their own [19]. While research led by foreign institutions brings valuable resources and expertise, it may not always prioritize culturally specific nuances and can face challenges in translating findings into local policy or practice. Therefore, there is a critical need for strengthening local research capacity within South Asia to ensure contextually relevant and impactful findings for its populations.

There are several factors that can help explain the observed trend. One key reason is that many of these foreign institutions are based in high-income countries, which typically have far greater funding, stronger research ecosystems, and larger international collaboration networks that enable higher research output [20]. In addition, sociocultural stigma surrounding drug use and mental health remains a major barrier in many South Asian countries [6], influencing not only treatment-seeking behavior but also research participation and topic prioritization. This stigma can lead to social desirability bias, underreporting of substance use, and deeply rooted taboos around discussing mental illness, all of which likely contribute to the low research output on addiction-related studies from these settings. In some of the countries, such as Nepal [21] and Bangladesh [22], policy frameworks often prioritize criminalization over treatment of addiction, creating further legal and logistical barriers for researchers. Language barriers also play a role, as many journals (over 93%) publish exclusively in English [23]. Researchers from countries with lower levels of English proficiency, such as Afghanistan and Bhutan, may face significant challenges in preparing manuscripts, leading to lower publication numbers from these regions.

Limitations

This study has several limitations that should be considered when interpreting the findings. First, we only used the PubMed database to identify all relevant research publications on substance addiction in South Asian populations. This may have omitted relevant publications from other major databases (e.g., Scopus, Web of Science, PsycINFO), resulting in underrepresentation of the measured research productivity in this field. In addition, our search strategy determined relevant publications using search terms from titles and abstracts of articles, which may have missed studies where South Asian populations were included but not explicitly mentioned in titles or abstracts. Furthermore, the country of publication was determined solely by the first author’s affiliation to indicate where the majority of the work was conducted, which risks misclassification in studies with multi-country collaborations. We also did not distinguish between in-region and diaspora South Asian populations in our analysis, as this information was often not reported, which limited our ability to compare research patterns across these groups. Lastly, this study excluded non-peer-reviewed or grey literature (e.g., technical reports, government publications, newspaper articles, and conference proceedings) when evaluating research productivity. However, these sources may be important indicators of research activity in low-resource South Asian communities, where valuable public health data are documented outside of indexed peer-reviewed journals.

## Conclusions

In summary, this bibliometric analysis demonstrates both encouraging progress and enduring disparities in substance addiction research focused on South Asian populations over the past decade. While overall publication output has significantly increased over the years, this growth was largely driven by studies on the Indian population, and the other groups remained vastly underrepresented relative to their population size or health burden. These underrepresented groups also had lower-quality research publications, and much of the existing work originated from institutions outside South Asia. Together, these findings underscore the need for a more balanced and inclusive research landscape-one that prioritizes underrepresented populations and strengthens locally led investigations in South Asia. To build on our findings, future studies should validate these patterns using larger datasets from multiple databases and should further differentiate between in-region and diaspora South Asian populations. Additionally, future bibliometric analyses could employ thematic mapping or keyword co-occurrence techniques to identify the major research topics in this field, such as whether certain substances (e.g., opioids, alcohol, cannabis) or barriers (e.g., stigma, cultural norms) dominate the discourse on South Asian substance addiction research. In doing so, this field can bridge critical knowledge gaps and guide the development of evidence-based interventions, ensuring that no population is left behind in addressing substance addiction.
